# Transitions at CpG Dinucleotides, Geographic Clustering of *TP53* Mutations and Food Availability Patterns in Colorectal Cancer

**DOI:** 10.1371/journal.pone.0006824

**Published:** 2009-08-31

**Authors:** Fabio Verginelli, Faraz Bishehsari, Francesco Napolitano, Mahboobeh Mahdavinia, Alessandro Cama, Reza Malekzadeh, Gennaro Miele, Giancarlo Raiconi, Roberto Tagliaferri, Renato Mariani-Costantini

**Affiliations:** 1 Department of Oncology and Neurosciences, “G. d'Annunzio” University, and Center of Excellence on Aging (CeSI), “G. d'Annunzio” University Foundation, Chieti, Italy; 2 Digestive Disease Research Center (DDRC), Shariati Hospital, University of Tehran, Tehran, Iran; 3 Department of Mathematics and Informatics, University of Salerno, Salerno, Italy; 4 Department of Physical Sciences, University of Naples, Naples, Italy; The University of Hong Kong, Hong Kong

## Abstract

**Background:**

Colorectal cancer is mainly attributed to diet, but the role exerted by foods remains unclear because involved factors are extremely complex. Geography substantially impacts on foods. Correlations between international variation in colorectal cancer-associated mutation patterns and food availabilities could highlight the influence of foods on colorectal mutagenesis.

**Methodology:**

To test such hypothesis, we applied techniques based on hierarchical clustering, feature extraction and selection, and statistical pattern recognition to the analysis of 2,572 colorectal cancer-associated *TP53* mutations from 12 countries/geographic areas. For food availabilities, we relied on data extracted from the Food Balance Sheets of the Food and Agriculture Organization of the United Nations. Dendrograms for mutation sites, mutation types and food patterns were constructed through Ward's hierarchical clustering algorithm and their stability was assessed evaluating silhouette values. Feature selection used entropy-based measures for similarity between clusterings, combined with principal component analysis by exhaustive and heuristic approaches.

**Conclusion/Significance:**

Mutations clustered in two major geographic groups, one including only Western countries, the other Asia and parts of Europe. This was determined by variation in the frequency of transitions at CpGs, the most common mutation type. Higher frequencies of transitions at CpGs in the cluster that included only Western countries mainly reflected higher frequencies of mutations at CpG codons 175, 248 and 273, the three major *TP53* hotspots. Pearson's correlation scores, computed between the principal components of the datamatrices for mutation types, food availability and mutation sites, demonstrated statistically significant correlations between transitions at CpGs and both mutation sites and availabilities of meat, milk, sweeteners and animal fats, the energy-dense foods at the basis of “Western” diets. This is best explainable by differential exposure to nitrosative DNA damage due to foods that promote metabolic stress and chronic inflammation.

## Introduction

The *TP53* gene (OMIM no. 191117), which encodes a tumor-suppressor protein that drives multiple cellular responses to stress, including cell-cycle arrest, DNA repair, apoptosis, metabolism and autophagy, is frequently mutated in cancer [1], [2], [3], [4], [5], [6]. *TP53* mutations are mostly missense and cluster in exons 5–8, the evolutionarily-conserved region of the DNA-binding domain that contains ≈90% of the known mutations and all mutation hotspots at CpG dinucleotides [7], [8], [9], [10], [11]. Laboratory models and data from tumors with established environmental risk factors show that *TP53* mutation patterns reflect primary mutagenic signatures of DNA damage by carcinogens, vulnerability of nucleotide positions in DNA secondary structure, efficiency of repair processing, and selection for loss of trans-activation properties [10], [11], [12], [13], [14], [15], [16].

Colorectal cancer (CRC), worldwide one of the most common malignancies, is mainly attributed to dietary risk factors [17], [18], [19], [20], [21], [22], [23], [24]. *TP53* mutations are found in 50-60% of all CRCs and are thought to originate in precancerous lesions, where aberrantly proliferating colonocyte progenitors are directly exposed to dietary residue [25], [26]. Nevertheless the *TP53* mutation pattern typical of CRC cannot be easily correlated to diet, because it is characterized by a striking preponderance of G:C>A:T transitions [9], [13], [16]. These are the most frequent base substitutions induced by reactive oxygen species, byproducts of normal aerobic metabolism generated at high levels in all inflammatory processes and after exposure to a wide variety of carcinogens and toxicants [27], [28], [29], [30], [31], [32], [33], [34]. Furthermore CRC development appears to depend on whole-life nutrition pattern [23], and *TP53* mutations may occur years before CRC diagnosis [25], [35]. Thus the time-frame for the estimation of diet may not fully capture the period relevant for mutagenesis and carcinogenesis. This is complicated by the relatively limited variation in dietary habits within single populations, by biases in reporting and recording dietary intakes and by the problematic assessment of exposures to food-borne carcinogens and toxicants, natural and generated in foods production, processing, preservation, and preparation [17], [23], [36], [37], [38], [39], [40], [41], [42]. Adding to complexity, intestinal mutagenesis may be modified by nutrient/nutrient, nutrient/microflora, nutrient/cell metabolism, nutrient/gene and nutrient/DNA repair interactions, and affected by epigenetic modifications, transit time of dietary residue, inflammatory and endocrine responses, body mass and energy consumption through physical activity [23], [40], [43], [44], [45], [46], [47], [48].

Geography strongly impacts on the ecological, cultural and economic factors that determine food systems and diets. CRCs from patients embedded in geographically diverse populations and cultures reflect substantially different dietary exposures, extended over the whole-life course and unbiased by estimation errors [17], [21], [23]. Thus food-related mutational signatures could be highlighted through the analysis of geographic variation in CRC-associated *TP53* mutations. To test such hypothesis, we analyzed 2,572 *TP53* mutations associated with primary CRCs from 12 countries or geographic areas. The mutations (Database S1) were extracted from the *TP53* database of the International Agency for Research on Cancer (IARC) (R10 update, July – 2005, http://www-p53.iarc.fr/Somatic.html), with the addition of an Iranian series [11], [49]. To investigate correlations between geographic clustering of *TP53* mutations and foods, we relied on the food balance sheets (FBS) of the Food and Agriculture Organization of the United Nations (FAO, http://faostat.fao.org/site/368/DesktopDefault.aspx?PageID=368), that provide unique comprehensive pictures of the patterns of national food supply, useful for international comparisons [50], [51], [52]. Food availability patterns (FPs) for the countries/geographic areas in the *TP53* database were derived from the mean *per caput* supplies, in percent of the total caloric value, of each major food group available for human consumption during the reference year 1990 [17] (Dataset S1). The datamatrices generated for mutation sites (MS), mutation types (MT) and FP (Datamatrices S1) were investigated for geographic variation by hierarchical clustering (HC). Factors underlying HC were defined by feature analysis (FA) through principal component analysis (PCA). Pearson's correlation scores were computed between the principal components of the mutation type, food availability pattern and mutation site datamatrices. These analyses demonstrated significant correlations between transitions at CpGs and both mutation sites as well as availabilities of meat, milk, sweeteners and animal fats. Our results could be best explainable with differential exposure to nitrosative DNA damage due to the consumption of energy-dense foods that promote metabolic stress and chronic low-grade inflammation.

## Results

### Geographic variation in mutation site and type

**Figure 1:**
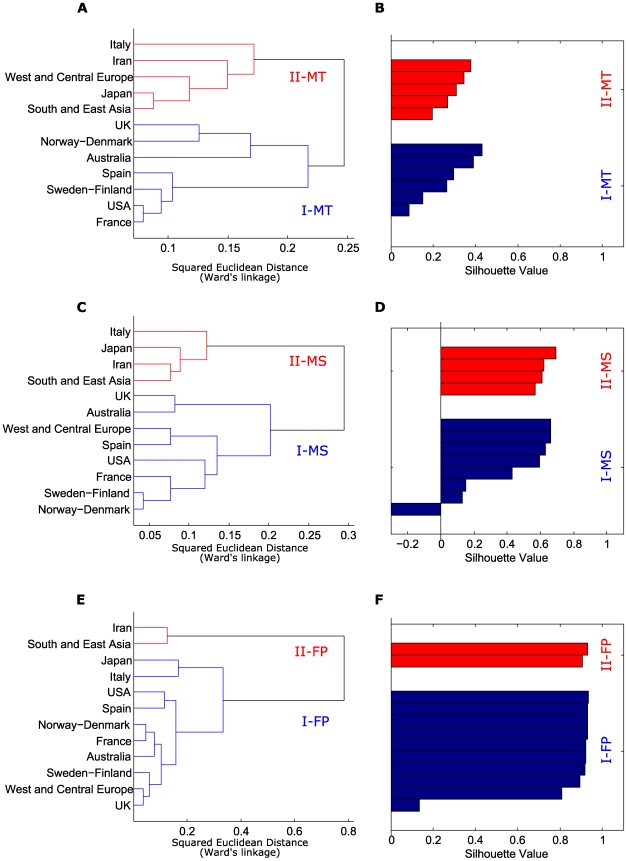
Hierarchical clusterings for *TP53* mutation sites, *TP53* mutation types and food patterns. Hierarchical clusterings (HC) by country/geographic area and silhouette plots for: A–B, *TP53* mutation sites (MS); C–D, *TP53* mutation types (MT); E–F, food patterns (FP).

**Figure 2:**
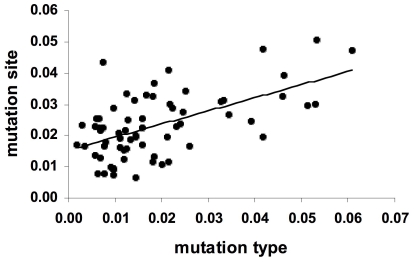
Mantel test correlation between *TP53* mutation sites and *TP53* mutation types. Mantel test shows correlation between the distance matrices of mutation sites (MS) and mutation types (MT), with regression line parameter: r = 0.581, c = 0.015; R^2^ = 0.338, *P* = 0.001 after 10,000 permutations.

Panels A–B and C–D of Figure 1 respectively show hierarchical clustering (HC) by country/geographic area for *TP53* mutation sites (MS), based on 2,542 exonic mutations, and types (MT), based on 2,572 mutations in exons and intron-exon boundaries. The MS and MT trees showed similar structures, each with two major geographic clusters, one including only Western countries (I-MS, I-MT), the other Asia and parts of Europe (II-MS, II-MT). The main difference consisted in the position of West and Central Europe in II-MS and I-MT, respectively. Stability of clusters was assessed by silhouette values. Silhouette plots for different thresholds, applied to each dendrogram, were compared to assess the reliability of the clustering solutions. In both cases the tree structure showed two stable clusters. The low silhouette value of MT was related to the poor stability of the “Spain” branch, attributable to either I-MT or II-MT. The MS and MT tree structures were correlated by two-tailed Mantel test (r = 0.581, *P* = 0.001) (Figure 2).

**Figure 3:**
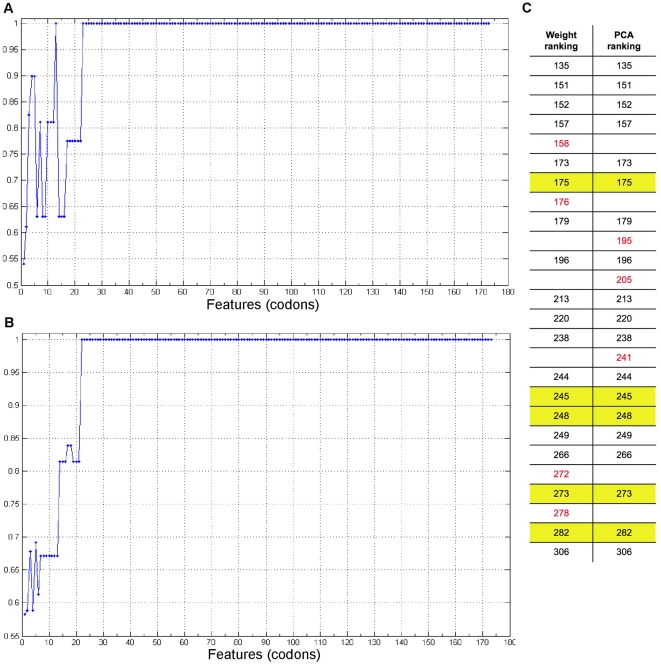
Feature selection by heuristic approach for the mutation sites data. Similarity values are on the y axis; number of features (*i.e.*, codons) on the x axis, respectively ranked in decreasing order of weight (number of mutations, panel A), and decreasing order of the first 11 PC coefficients of each feature (where 11 was the number of PCs contributing 100% of the data variance, panel B). Panel C lists (in order of number) the codons (features) with highest variance in the mutation sites (MS) data, selected by weight ranking (23/173 codons) and by PC coefficients ranking (22/173 codons). Overall 19 codons, including the five *TP53* mutation hotspots (highlighted in yellow), were selected by both methods.

By multivariate FA we next investigated the factors that determined clustering for MS (*i.e*., codons) and MT (*i.e*., mutation types), respectively using heuristic or exhaustive approaches. Feature selection aimed at identifying the minimum subset of features necessary to generate the clustering structure obtained using all the features. Sequential forward feature selection with two different rankings, respectively based on the number of mutations recorded for each codon (feature) and on the PC coefficients of each feature, was used to analyze the MS datamatrix by heuristic approach (Figure 3).

**Figure 4:**
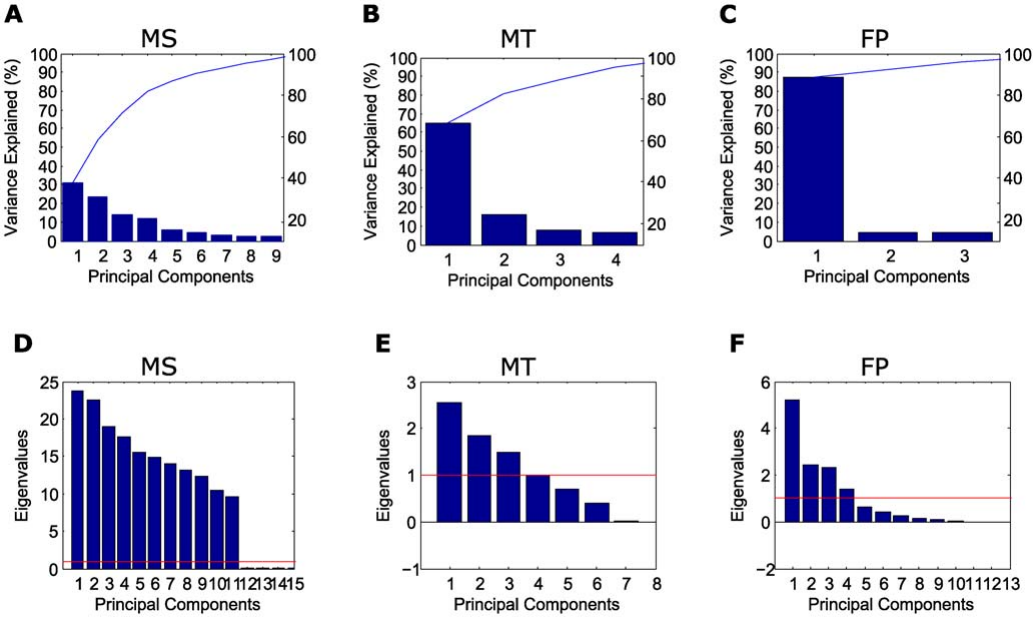
Scree and Kaiser's tests applied to the analysis of variance by PCA of the datamatrices for mutation sites, mutation types and food patterns. Results obtained using Scree test are shown in panels A–C. Total mutation sites (MS) variance (panel A) is explained by 11 components, of which only 9 are visualized, being the Scree test cut at the 98% level. Four components contribute 80% of the MS variance, the first accounting for 31%. Total mutation types (MT) variance (B) is explained by 4 components, the first of which, by far the most relevant, contributes 65% of variance. Total food patterns (FP) variance (C) is explained by 3 components, the first of which contributes 87.3% of variance. Results obtained using Kaiser's test are shown in panels D–F. The first 11 PCs for mutation sites (MS) (A), the first 3 PCs for mutation types (MT) (B) and the first 4 PCs for food patterns (FP) (C) have eigenvalues above 1 (red line).

Stable MS clustering was obtained with 23 weight-ranked or 22 PCA-ranked codons, in both cases including the five *TP53* mutation hotspots (*i.e*., CpG codons 175, 245, 248, 273, 282) [9], [13], [16], out of 173 mutated codons in the datamatrix. The variance contributed by the PCs of the MS datamatrix and their eigenvalues are shown in panels A and D of Figure 4, respectively. Total MS variance was explained by 11 components. Four components contributed 80% of the variance, and the first component, which accounted for 31%, had highly significant PC coefficients for the features corresponding to the five CpG hotspots, as detailed in File S1 and in Figure S1, panel A.

Exhaustive multivariate FA of the MT datamatrix is reported in Tables 1 and 2. In decreasing order, the most relevant features were G:C>A:T at CpGs, followed by A:T>C:G^,^ G:C>A:T and G:C>C:G. The variance contributed by each PC of the MT datamatrix and their eigenvalues are shown in panels B and E of Figures 4 respectively. Total MT variance was explained by 4 components, the first of which accounted for 65%, and, as detailed in File S1 and in Figure S1, panel D, the highest PC feature loading among the 8 mutation types corresponded to transitions at CpGs. Other mutations, including transitions at non-CpGs, were associated to minor fractions of variance.

**Table 1:** Worst-case exhaustive feature analysis of the mutation types datamatrix.

|S| = number of selected features (j)	1	2	3	4	5	6	7	8
Feature (i)								
**1. A:T>C:G**	0.42	0.39	0.32	0.38	0.31	0.38	0.38	1.00
**2. A:T>G:C**	0.49	0.44	0.34	0.39	0.31	0.39	0.38	1.00
**3. A:T>T:A**	0.44	0.40	0.32	0.40	0.38	0.38	0.38	1.00
**4. FS**	0.50	0.40	0.34	0.33	0.31	0.38	0.38	1.00
**5. G:C>A:T**	0.42	0.40	0.37	0.33	0.38	0.38	0.38	1.00
**6. G:C>A:T at CpG**	**0.92**	**0.80**	**0.80**	**0.79**	**0.77**	**0.80**	**0.81**	**1.00**
**7. G:C>C:G**	0.36	0.37	0.32	0.33	0.31	0.38	0.38	1.00
**8. G:C>T:A**	0.51	0.37	0.37	0.33	0.31	0.38	0.38	1.00

Worst similarity values for each subset S of the MT features, including the selected feature in the left column, are computed using the exhaustive multivariate feature analysis. Bold characters highlight the worst similarity values of the features that most influence cluster structure. Entry (i,j) reports the minimum similarity value obtained using the i-th feature together with any other j-1 features. For example, feature A:T>C:G gives a similarity value that is at least 0.38 when coupled with any 6 of the features in the left column.

**Table 2:** Best-case exhaustive feature analysis of the mutation types datamatrix.

Column 1(Feature)	Column 2 (Features that paired with feature in column 1 give the same clustering obtained with all features)
A:T>C:G	G:C>A:T at CpG
A:T>G:C	none
A:T>T:A	none
FS	none
G:C>A:T	G:C>A:T at CpG
G:C>A:T at CpG	A:T>C:G and/or G:C>A:T and/or G:C>C:G
G:C>C:G	G:C>A:T at CpG
G:C>T:A	none

Exhaustive multivariate feature analysis is used to highlight pairs of MT features giving similarity value equal to 1. Features in column 1 give similarity 1 if and only if coupled with one of the features in column 2. For example, feature A:T>C:G gives a similarity value 1 when coupled with G:C>A:T at CpGs. Relevance of individual features in column 1 is based on the number of other features in column 2 with which it can yield unitary value after pairing. The most relevant feature is G:C>A:T at CpGs, followed by A:T>C:G^,^ G:C>A:T and G:C>C:G.

**Figure 5:**
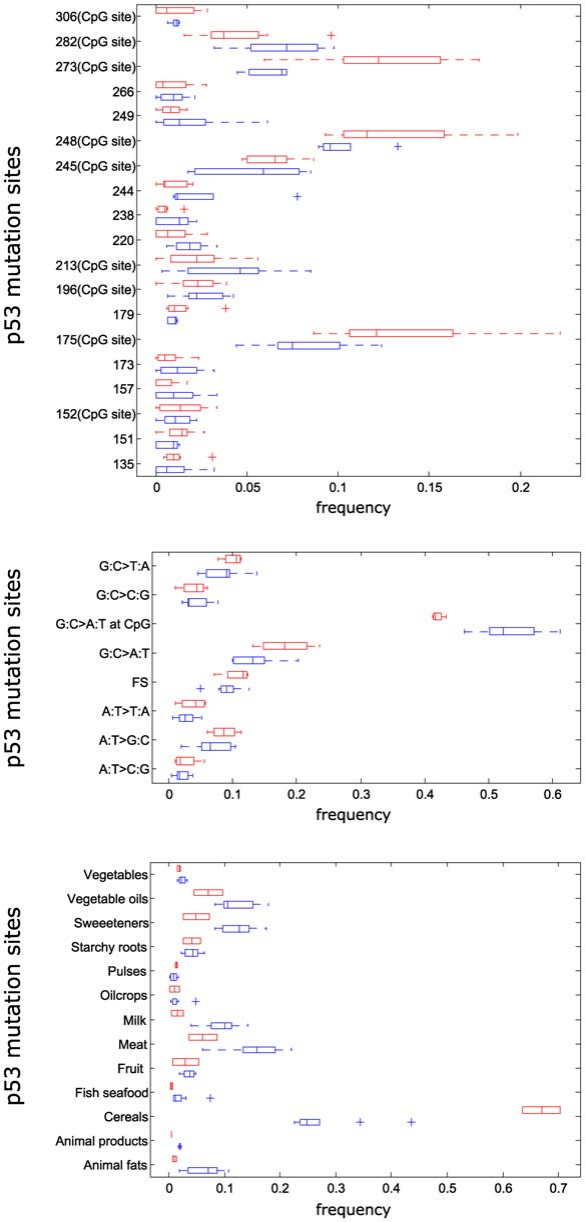
Box-plots of feature relevance for mutation sites, mutation types and food patterns. In each panel, box plots pertaining to clusters I versus II of mutation sites (MS), mutation types (MT) and food patterns (FP) obtained by hierarchical clustering are color-coded in red (cluster I) and blue (cluster II), respectively. Panel A: frequency box-plots of mutations at the 19 codons with highest weights and highest PCA variance coefficients (identified in Figure 4). Higher mutation frequencies at the three major CpG hotspot codons 175, 248, and 273 in I-MS versus II-MS are evident. Panel B: frequency box-plots of the 8 mutation types, showing higher frequencies of transitions at CpGs in I-MT (range: 46.1–61.2%) versus II-MT (range: 41.2–43.3%). Panel C: box-plots of the mean percent of the total available caloric value from each major food group in the relevant countries/geographic areas, showing lower cereals for the countries/geographic areas in I-FP versus those in II-FP, balanced by higher meat, milk, sweeteners and animal fats.

**Figure 6:**
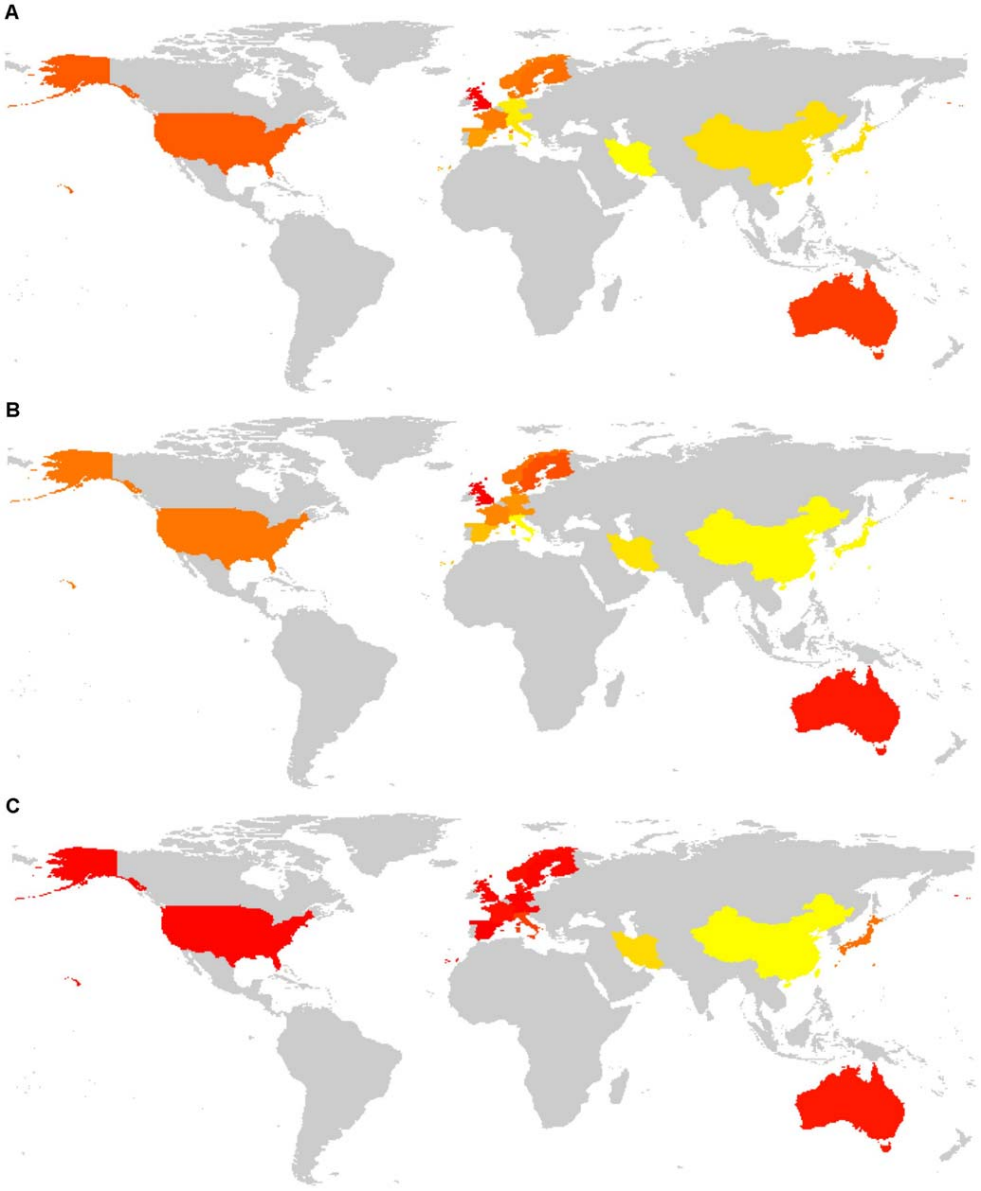
Geographic visualization of the most relevant features evidenced in the box-plots. Selected features of the mutation sites (MS, A), mutation types (MT, B) and food availability patterns (FP, C) datamatrices were mutations at the three major *TP53* hotspot codons 175, 248, and 273 for MS; G:C>A:T mutations at CpGs for MT; meat/milk/sweeteners/animal fats (added), cereals (subtracted) for FP. Feature frequencies were summed and projected in yellow to red color range onto the geographic profiles of the relevant countries/geographic areas.

The frequency box-plots of the mutations at the 19 codons with highest weights and highest PCA variance coefficients in Figure 5, panel A, showed higher mutation frequencies at the three major hotspot codons 175, 248, and, particularly, 273, in I-MS versus II-MS. This reflected higher frequencies of transitions at CpGs in I-MT (range: 46.1–61.2%) versus II-MT (range: 41.2–43.3%) in the frequency box-plots of the 8 mutation types in Figure 5, panel B. Such most relevant features were used to geographically visualize MS and MT variation (Figure 6, panels A–B). Highlighted groupings of countries/geographic areas were similar to the MS and MT clusters in Figure 1, obtained by HC using all the features. Overall these results indicate that in CRC *TP53* transition mutagenesis at CpGs is modulated by geography-related factors. This might reflect differences in exposure(s) to specific food-associated mutagenic process(es) [53].

### Geographic variation in food supply patterns

To address this issue, we analyzed the FP datamatrix by HC and FA through PCA. HC for FP was based on the mean *per caput* supply values, in percent of total available calories, of each major food group in the relevant countries/geographic areas during the reference year 1990 [17]. HC yielded two major clusters, I-FP, with Western countries and Japan, and II-FP, with South and East Asia plus Iran (Figure 1, panels E–F). The clusterization of Japan in I-FP had a low silhouette value and contrasted with the previous assignments of Japan to clusters II-MS and II-MT. To verify Japan's assignment, we generated all the possible subsets of the 13 FP features (food groups), *i.e*., 8,192 subsets. HC trees, cut to obtain two clusters, were then generated based on each of these subsets. Dendrograms were classified as A or B when Japan clusterized respectively in II-FP or I-FP, and as C, when different from A and B. Overall 2,405 clusterings, classified as A, assigned Japan to cluster II-FP with Iran and South and East Asia; 4,178, classified as B, assigned Japan to cluster I-FP, with Western countries; and 1,609 were classified as C, being different from A and B. The histograms in Figure S2, panels A–B, that visualize the number of times that each of the 13 features was present when type A or B clusterings respectively were obtained, readily show that feature cereals was almost always absent in type A clusterings and almost always present in type B clusterings. Thus Japan joined I-FP only because of the low availability of cereals.

Tables 3 and 4 show the results of exhaustive FA of the FP datamatrix. In decreasing order, the most relevant features were cereals, milk, and meat. PCA showed that total FP variance was explained by 3 components, the first of which accounted for a major fraction of 87.3% (Figure 4, panels C and F). The variance of this component, which, in loading order, included the features cereals, meat, milk, sweeteners, animal fats (File S1 and Figure S1, panel G), explained the tree structure, determined by lower cereals and higher meat, milk, sweeteners and animal fats in I-FP relative to II-FP, as shown in panels C and F of Figure 4.

**Table 3:** Worst-case exhaustive feature analysis of the food patterns datamatrix.

|S| = number of selected features (j)	1	2	3	4	5	6	7	8	9	10	11	12	13
Feature (i)													
**1. Animal fats**	0.53	0.43	0.43	0.45	0.54	0.54	0.54	0.54	0.54	0.71	1.00	1.00	1.00
**2. Animal products**	1.00	0.51	0.51	0.51	0.51	0.53	0.53	0.54	0.54	0.71	1.00	1.00	1.00
**3. Cereals**	**1.00**	**1.00**	**1.00**	**1.00**	**1.00**	**1.00**	**1.00**	**1.00**	**1.00**	**1.00**	**1.00**	**1.00**	**1.00**
**4. Fish/seafood**	0.47	0.34	0.32	0.36	0.40	0.53	0.53	0.54	0.54	0.71	1.00	1.00	1.00
**5. Fruit**	0.65	0.52	0.52	0.51	0.49	0.53	0.53	0.54	0.54	0.71	1.00	1.00	1.00
**6. Meat**	0.71	0.71	0.71	0.71	0.71	0.71	0.72	0.72	0.72	0.80	1.00	1.00	1.00
**7. Milk**	1.00	0.80	0.71	0.71	0.71	0.71	0.71	0.71	0.71	0.71	1.00	1.00	1.00
**8. Oilcrops**	0.55	0.37	0.39	0.36	0.40	0.53	0.53	0.54	0.54	0.71	1.00	1.00	1.00
**9. Pulses**	0.40	0.40	0.35	0.36	0.40	0.53	0.53	0.54	0.54	0.71	1.00	1.00	1.00
**10. Starchy roots**	0.40	0.31	0.32	0.36	0.40	0.53	0.53	0.54	0.54	0.71	1.00	1.00	1.00
**11. Sweeteners**	0.58	0.56	0.54	0.54	0.54	0.54	0.54	0.54	0.54	0.71	1.00	1.00	1.00
**12. Vegetable oils**	0.51	0.51	0.51	0.51	0.51	0.54	0.54	0.70	0.70	1.00	1.00	1.00	1.00
**13. Vegetables**	0.35	0.31	0.32	0.49	0.40	0.53	0.53	0.54	0.54	0.71	1.00	1.00	1.00

Worst similarity values for each subset S of the FP features, including the selected feature in the left column, are computed using the exhaustive multivariate feature analysis. Bold characters highlight the worst similarity values of the features that most influence cluster structure (cereals). Entry (i,j) reports the minimum similarity value obtained using the i-th feature together with any other j-1 features. For example, feature “animal products” (i = 2) gives a similarity at least equal to 0.71 combined with any other remaining 9 features (j = 10).

**Table 4:** Best-case exhaustive feature analysis of the food patterns datamatrix.

Column 1 (Feature)	Column 2 (Features that paired with feature in column 1 give the same clustering obtained with all features)
Animal fats	Cereals, Meat, Milk
Animal products	Cereals, Meat, Oilcrops, Vegetable Oils
Cereals	All
Fish/Seafood	Cereals, Meat,Milk
Fruit	Cereals, Milk
Meat	Animal fats, Cereals, Fish/Seafood, Milk, Oilcrops, Starchy roots, Vegetable oils
Milk	All but Sweeteners
Oilcrops	Animal Products, Cereals, Meat, Milk
Pulses	Cereals, Milk
Starchy roots	Cereals, Meat, Milk
Sweeteners	Cereals
Vegetable oils	Cereals, Meat, Milk
Vegetables	Animal products, Cereals, Milk

Exhaustive multivariate feature analysis is used to highlight pairs of FP features giving similarity value equal to 1. Features in column 1 give similarity equal to 1 if and only if coupled with one of the features in column 2. For example, feature cereals (i = 3) gives a similarity that is always equal to 1 alone or together with any set of other features. Relevance of individual features in column 1 is based on the number of other features in column 2 with which it can yield unitary value after pairing. The most relevant features are cereals, milk, and meat.

### Correlations between mutation pattern and food supply pattern

The data from the MS, MT and FP datamatrices were projected on the 1-dimensional space spanned by their respective PCs. Pairwise Pearson correlations were then computed for the three datamatrices in all the projected spaces. Tables 5 to [Table pone-0006824-t006] 7 show the correlation scores, and the corresponding *P*-values, obtained for the first 3 PCs of each datamatrix, that, except for MS, accounted for most of the variance. Pearson's correlation between the PCs for MT and for FP (Table 5) showed that the first PC for MT was correlated with the first PC for FP, with r = −0.60 (*P* = 0.039). Availabilities of meat, milk, sweeteners and animal fats were directly correlated to transitions at CpGs, availability of cereals to transitions at non-CpGs (File S1 and Figure S1, panels D and G). As detailed in File S1 and in Figure S1, other less important correlations involved second and third PCs that accounted for minor fractions of variance. With the same analysis, the first PCs for MS and for MT resulted again strongly correlated, with r = −0.87 (*P* = 0.0002, Table 6), which supported Mantel's test results (Figure 2). However, in spite of the correlation between MT and FP, there were no significant correlations between the PCs of MS and FP (Table 7).

**Table 5:** Pearson's correlation scores between the PCs of mutation types and food patterns.

PCs	1^th^ PC (MT)	2^nd^ PC (MT)	3^rd^ PC (MT)
**1^th^ PC (FP)**	−0.6003* [*0.0391*]	0.2534 **[** *0.4268* **]**	0.6194* [*0.0317*]
**2^nd^ PC (FP)**	−0.6021* [*0.0383*]	−0.1735 **[** *0.5897* **]**	−0.4654 **[** *0.1273* **]**
**3^rd^ PC (FP)**	0.1724 **[** *0.5921* **]**	0.6050* [*0.0371*]	−0.1058 **[** *0.7434* **]**

Pearson's correlation scores computed between the principal components (PC) of mutation types (MT) and food availability patterns (FP), with the corresponding *P*-values (square brackets). Significant correlations are highlighted in bold and the corresponding r-coefficient values are marked with an asterisk (*).

**Table 6 pone-0006824-t006:** Pearson's correlation scores between the PCs of mutation types and mutation sites.

PCs	1^th^ PC (MT)	2^nd^ PC (MT)	3^rd^ PC (MT)
**1^th^ PC (MS)**	−0.8742* [*0.0002*]	−0.0908 **[** *0.7790* **]**	−0.0118 **[** *0.9709* **]**
**2^nd^ PC (MS)**	0.0975 **[** *0.7630* **]**	−0.1414 **[** *0.6612* **]**	−0.0568 **[** *0.8609* **]**
**3^rd^ PC (MS)**	−0.0069 **[** *0.9830* **]**	0.2340 **[** *0.4642* **]**	0.3828 **[** *0.2194* **]**

Pearson's correlation scores computed between the principal components (PC) of mutation types (MT) and mutation sites (MS), with the corresponding *P*-values (square brackets). Significant correlations are highlighted in bold and the corresponding r-coefficient values are marked with an asterisk (*).

**Table 7:** Pearson's correlation scores between the PCs of mutation sites and food patterns.

PCs	1^th^ PC (MS)	2^nd^ PC (MS)	3^rd^ PC (MS)
**1^th^ PC (FP)**	0.4176 [*0.1767*]	−0.4246 [*0.1688*]	0.4168 [*0.1777*]
**2^nd^ PC (FP)**	0.3858 [*0.2155*]	0.1226 [*0.7042*]	−0.2474 [*0.4382*]
**3^rd^ PC (FP)**	−0.2826 [*0.3735*]	−0.4014 [*0.1960*]	−0.1824 [*0.5705*]

Pearson's correlation scores computed between the principal components (PC) of mutation sites (MS) and food availability patterns (FP), with the corresponding *P*-values (square brackets). Significant correlations are highlighted in bold and the corresponding r-coefficient values are marked with an asterisk (*).

**Figure 7:**
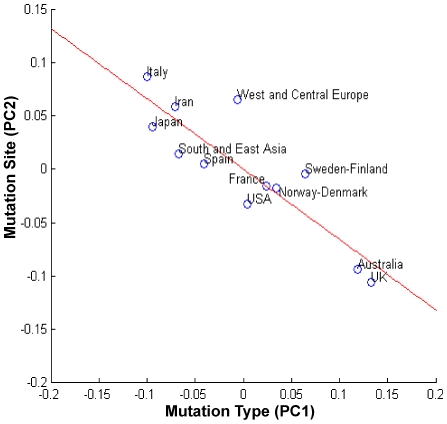
Scatter plot of the correlation between mutation sites and types according to countries/geographic areas. Scatter plot of the countries/geographic areas in the *TP53* database projected on the 2-dimensional space spanned by the first principal components of mutation sites (Mutation Site PC2) and mutation types (Mutation Type PC1). Italy, Iran, South and East Asia and West and Central Europe have relatively lower frequencies of mutations at CpG hotspot codons, compensated by higher frequencies of mutations at all other sites (see also box-plots in Figure 5). Mutation frequencies at CpG hotspots increase in other countries, with highest frequencies in Australia and UK. A linear regression shows the global trend of the correlation (r = −0.8742).

**Figure 8:**
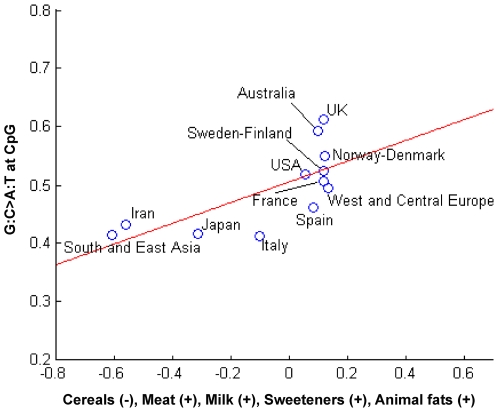
Scatter plot of the correlation between mutation types and food patterns according to countries/geographic areas. Scatter plot of the countries/geographic areas in the *TP53* database projected on the 2-dimensional space spanned by the highest coefficient features of the first principal component (PC) of food availability pattern (FP), *i.e*., cereals, meat, milk, sweeteners, animal fats, and of the first PC of mutation type (MT), *i.e*., G:C>A:T at CpGs. A linear regression shows the global correlation trend.

Scatter plots with superimposed linear regression showing the global trend of correlations were built for the countries/geographic areas as projected on the 2-dimensional spaces spanned by the first PCs of MS and MT (Figure 7) and of MT and FP (Figure 8). As shown in Figure 7, Italy, Iran, South and East Asia and West and Central Europe had relatively lower frequencies of mutations at CpG hotspot codons, compensated by higher frequencies of mutations at all other sites (see also box-plots in Figure 2). Mutation frequencies at CpG hotspots increased in other countries, with highest frequencies in Australia and UK. As shown in Figure 8, transitions at CpGs correlated with countries/geographic areas characterized by higher availabilities of energy-dense, Western-style foods, while South and East Asia, Iran, Japan and, to a lesser extent, Italy, where cereals were higher and meat, milk, sweeteners and animal fats lower, had lower frequencies of such mutations.

Overall, variation in the frequency of transitions at CpGs reflected variation in the availabilities of the energy-dense foods that form the basis of “Western-style” diets and that are linked to overweight and obesity [18], [20], [21], [22], [23]. Transitions at non-CpGs balanced decreases in transitions at CpGs in the countries/geographic areas where cereals compensated for lower availabilities of such foods.

## Discussion

Several studies addressed the issue of CpG transition mutagenesis in cancer, with particular regard to *TP53* mutations in CRC. Being exonic CpGs constitutively hyper-methylated, C to T mutations at coding CpGs in *TP53* should be scored as direct transitions from hypermutable 5-methylcytosine to thymine [54], [55], [56], [57], [58], [59]. Dietary folate is a defined environmental determinant of genomic methylation [23], [60], [61]. Laboratory models and data on CRCs in patients carrying a germline methylenetetrahydrofolate reductase (*MTHFR*) gene variant that results in reduced plasma and serum folate suggest that low folate, by inducing global hypomethylation, may decrease *TP53* transition mutagenesis at CpGs [62], [63], [64]. Folate-rich foods include fresh vegetables, pulses (legumes) and relatively unprocessed cereals [65], [66]. Little is known about DNA methylation variation among individuals and populations [67], [68]. We did not find any correlation between availability of vegetables or pulses and *TP53* mutation pattern, while cereals, relatively unprocessed in most Asian countries [69], [70], inversely correlated with transitions at CpGs. Thus folate availability may not account for our results. This conclusion agrees with studies showing that, in absence of interacting genetic effects, folate alone does not influence *TP53* mutation patterns in CRC (although it may affect TP53 protein expression) [44], [71], [72].

The hypermutability of endogenous 5-methylcytosine does not *per se* explain the unique role of transitions at CpGs in geographic clustering of *TP53* mutations [57], [58], [59]. However transitions at CpGs in *TP53* are efficiently induced by nitrosative DNA damage [31], [58], [59], [73], [74], [75]. Nitric oxide (NO), a critical signalling molecule implicated in the regulation of peristalsis, gut vasomotor functions and mucosal inflammation, may contribute to transition mutagenesis at CpGs acting directly at 5-methylcytosines, by nitrosative deamination in oxidizing environments, and, indirectly, at guanines, by base alkylation after conversion to nitrate, bacterial reduction to nitrite and endogenous formation of N-nitroso compounds [73], [74], [75], [76], [77], [78] [79] [80] [81] [82]. Mutagenesis at CpGs may be facilitated by NO-induced inhibition of DNA repair [75], [80]. Furthermore, NO promotes apoptosis via *TP53* and therefore exerts a critical selective pressure for *TP53* mutation [83], [84], [85], [86].

NO is produced at mutagenic concentrations by inducible NO synthase (iNOS), the widespread enzyme isoform upregulated by inflammatory cytokines [76], [82], [87]. It has already been suggested that the excess of *TP53* transitions at CpGs found in cancers arising on a chronic inflammatory background, such as CRC in ulcerative colitis and bladder cancer associated with *Schistosomiasis*, results from nitrosative stress [74], [88]. Moreover transitions at CpGs are strongly related to iNOS expression in both CRC and adenocarcinoma of Barrett's esophagus [89], [90]. Arginine, the substrate for NO synthesis and a potential CRC-related dietary factor [87], [91], [92], [93], is contained in a variety of protein-rich foods of animal and vegetable origin [65], [66] and may not *per se* explain why variation in the frequency of transitions at CpGs correlated with variation in the availabilities of meat, milk, sweeteners and animal fats. However it is known that these energy-dense foods promote a pro-inflammatory milieu that increases iNOS expression and NO production [23], [78], [94], [95], [96], [97], [98], [99], [100], [101]. In addition red meat is a major exogenous source of nitrogen compounds and haem, which contribute to N-nitrosation in the intestinal environment [23], [102], [103], [104], [105], [106], [107], [108]. Such considerations are supported by the fact that our data point to a key role of the ubiquitously methylated major *TP53* hotspot codons 175, 248 and 273 in geographic clustering. In fact, the vast majority of the mutations at these 3 codons reported in human cancer are compatible with nitrosative deamination [9], [11], [32], [54], [74], [109]. Moreover, transitions at codon 248 were experimentally induced with an NO-releasing compound [110] while mutations at codon 273 were found to be strongly associated with diets high in red meat and fat [44].

In conclusion, we recognize the difficulties inherent in interpreting causes and mechanisms responsible for CRC-associated *TP53* mutations, which are the end result of complex cascades of events. It is important to keep in mind the limitations of our analyses, based on a single, albeit large, database of mutations. Furthermore FAO FBS, the only standardized comprehensive food data available for international comparisons, approximate food supply patterns. Nevertheless, geographic variation in CRC-associated *TP53* mutation patterns appears to be due to transitions at CpGs and mainly related to differential mutation frequencies at the major *TP53* hotspots. This could be explainable by differential exposure to nitrosative DNA damage, linked to the consumption of foods promoting metabolic stress and chronic low-grade inflammation.

## Materials and Methods

### Databases, Datasets and Datamatrices

We analyzed 2,572 mutations in *TP53* exons 5–8 retrieved from primary CRCs, including 2,475 from 12 countries or geographic areas, extracted from the *TP53* database of the International Agency for Research on Cancer (IARC) (R10 update, July 2005, http://www-p53.iarc.fr/Somatic.html), and 97 from Iran [11], [49]. Mutations in adenomas, metastatic CRC and cell lines were excluded, as their spectrum could differ from that of primary CRC [111]. Analyses were based on 2,542 mutations in coding regions for MS, and on 2,572 (*i.e*., all) mutations for MT (Database S1). Mutations were grouped according to country or geographic area, the latter including geographically and ethnically related countries with low mutation numbers. Countries and number of mutations for MS and MT, were: Australia (including 6 mutations from New Zealand), MS:302, MT:302; USA, MS:233, MT:237; France, MS:215, MT:221; Italy, MS:181, MT:182; Spain, MS:181, MT:182; UK (including 3 mutations from Ireland), MS:131, MT:134; Iran, MS:94, MT:97; Japan, MS:323, MT:326. Geographic areas were: West and Central Europe (Germany, Austria, Switzerland, The Netherlands, Luxembourg), MS:174, MT:178; South and East Asia (China, Hong Kong, Taiwan, Singapore), MS:315, MT:318; Norway-Denmark, MS:162, MT:162; Sweden-Finland, MS:231, MT:233. Mutations from Brazil, Chile, Israel, Turkey, Korea and Eastern European countries listed in the R10 update of the IARC *TP53* database were excluded because of low numbers.

The FP dataset (Dataset S1) was extracted from the FAO FBS [50], [51] compiled for the reference year 1990 (http://faostat.fao.org/site/368/DesktopDefault.aspx?PageID=368), as used in reference [17]. Year selection tended to exclude the most recent and current international variations in food availabilities and nutrition, as CRC develops over several years and is mostly diagnosed in patients aged 65 years or older [112], while the IARC *TP53* database compiles mutations since 1989 [11]. The FP dataset included the following major food groups: animal fats, animal products, cereals, fish/seafood, fruit, meat, milk, oilcrops, pulses (legumes), starchy roots, sweeteners, vegetable oils and vegetables. For the purpose of this study alcohol was excluded, being much of the data on average availability of alcoholic drinks not informative and potentially confounding, due to large inter-individual variability [23]. Spices and stimulants, which account for low percentages of the total available daily energy supply, were also excluded. Statistical analyses were therefore conducted using the estimated percent (%) contribution of each considered food group to mean *per caput* daily energy availability [17]. Weighted average availabilities were calculated for geographic areas by adjusting for the 1990 population size of each included country. The MS, MT and FP datamatrices were normalized converting absolute numbers into frequencies (Datamatrices S1).

All standard techniques, including hierarchical clustering (HC), principal components analysis (PCA), Pearson correlation and linear regression, were used in their implementations from Matlab (2007b, The Mathworks and Matlab Statistics Toolbox).

### Statistical Pattern Recognition

Statistical pattern recognition allowed the integrated analysis of the MS, MT, and FP datamatrices to investigate relations between *TP53* mutation sites, *TP53* mutation types, and food supply patterns. The first analytical step consisted in clustering the 12 analyzed coutries/geographic areas by HC with respect to the data contained in the MS, MT and FP datamatrices. The stability of the obtained clusterings was assessed using the silhouette values. The similarities between the obtained clustering solutions, represented by dendrograms, were assessed using an entropy-based similarity measure. Feature analysis and selection, which is the process of studying the contribution of single features, or subsets of features, to dataset properties, was the next relevant processing step. Exact feature analysis can be performed testing the ability of each single subset of features to maintain a chosen property. In practice, this is feasible only when the number of features is low. In comparing the MT and FP datasets, because of the relatively low number of features, such exhaustive analysis could be carried out. With regard to the MS dataset, the number of possible subsets of features was too high, and therefore a heuristic approach, *i.e*., sequential forward selection, was used to select feature subsets. The principal components and the relative weights of the features were used as ranking criteria. Results were visualized on geographic maps with the relevant areas colored according to the most relevant features. Finally, multivariate correlations between the datasets were computed exploiting their PC projections. All these analytical steps are detailed below.

### Hierarchical clustering

Distance matrices for MS, MT and FP were computed by pairwise comparison between *TP53* countries/geographic areas using the squared Euclidean distance. Dendrograms were constructed through Ward's hierarchical clustering algorithm [113]. Stability of clusters was assessed evaluating the silhouette values [114] that measure how close each point in one cluster is to the points in the neighboring clusters. This measure ranges from +1, indicating points very distant from neighboring clusters, through 0, indicating points not distinctly in one cluster or another, to −1, indicating points probably assigned to the wrong cluster.

Matrices for MS, MT and FP were tested for correlation by Mantel's test [115]. The program Mantel version 3.1 was used to estimate Pearson correlation coefficients. Significance was assessed by 10,000 random permutations.

### Feature selection

Feature selection involved the use of a similarity measure between hierarchical clusterings, visualized as dendrograms, respectively built on the entire feature set and on the feature subset(s) to be tested. The higher the similarity, the higher the rank of the chosen feature subset. The entropy-based similarity measure used is defined below.

Two clusterings are identical if there is one-to-one correspondence between their clusters. The more a cluster of one clustering is filled with objects from different clusters of the other clustering (disorder), the less is the concordance between clusterings. All the information needed to summarize this phenomenon is the corresponding confusion matrix. Given two clusterings, 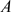 and 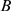 **,** where 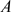 is made of 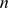 clusters and 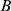 of 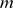 clusters, the confusion matrix 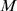 between 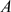 and 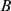 is an 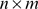 matrix, in which the entry 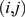 reports the number of objects in the cluster 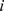 of 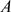 falling into the cluster 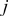 of 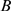. Entropy is the obvious tool to measure such disorder. If 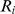 is the 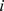-th row of 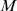 and 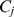 is the 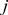-th column of 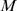, then 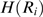 measures the disorder of the 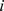-th cluster of 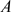 with respect to 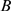, and 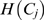 measures the disorder of the 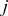-th cluster of 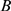 with respect to 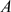.

A way to compute the similarity between 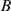 and 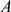 is the mean entropy of the clusters of 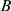 versus 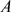, where the *a priori* probability of a cluster 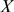, 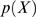, can be approximated as 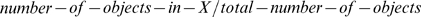, giving the formula:
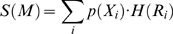
expressing the similarity of 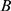 versus 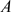, while the similarity of 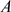 versus 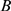 can be obtained with the analogue formula on 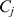, which turns to be 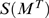. The measure of similarity between clusterings is in the trade-off between 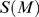 and 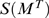. We define the final similarity measure:
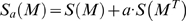
where 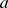, 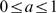, can be used to set the acceptable level of ‘sub-clusteringness’ of 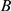 with respect to 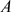. When 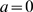, no importance is given to the fragmentation level of the clusters in 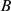. When 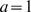 only exact matching between 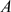 and 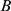 will produce a maximum for 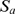.

Basing on such similarity measure between clusterings, useful comparisons between dendrograms can be easily performed. Given a solution obtained from a dendrogram (the target solution), it is possible to assess how much such solution can be approximated by another dendrogram.

Given a dendrogram 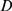, let 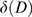 be the clustering solution obtained applying a cutting threshold 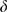 to 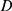. We define *complete threshold set* for a dendrogram any minimal set of threshold values, applying which all the possible clusterings for the dendrogram can be obtained. We indicate any such set for a dendrogram 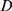 by 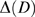. It can be easily shown that 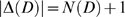, where 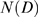 is the number of nodes in 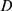.

Given a dendrogram 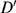, a target solution 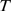 can be derived applying a cutting threshold. The similarity between 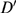 and another dendrogram, 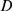, can be approximated using the dendrogram similarity procedure.

Dendrogram similarity procedure 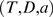


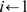
for each 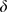 in 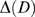


Build 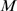, the confusion matrix between 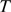 and 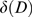


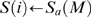


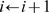
return 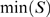


### Exhaustive approach to feature selection for the MT and FP datamatrices

Feature analysis studies the properties of single features or subsets of features of the analyzed data. Exact feature analysis can be performed testing the properties of each possible subset of features. In this study, the property of interest was the ability to maintain the groups obtained in the clustering analysis phase. Such exhaustive approach was successfully performed on the MT and FP datamatrices.

Given a set of features 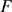 and a scoring function 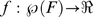, the exhaustive feature analysis approach consists in computing 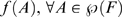. We performed this analysis using the features of the MT and FP datamatrices in turn for 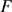 and 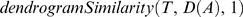 for 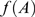, where T is the solution obtained in the clustering analysis phase of the data and 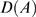 is the dendrogram built using the features subset 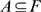.

The results of exhaustive feature analysis are reported in Tables 1 and 2 for the MT datamatrix and in Tables 3 and 4 for the FP datamatrix. In Tables 1 and 3 the entry 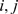 reports the worst score obtained using 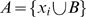, where 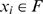 and 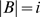. In Tables 2 and 4 the 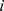 *-th* entry reports the set of features 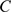 such that 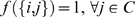.

### Heuristic approach to feature selection for the MS datamatrix

Being the number of MS feature subsets equal to 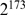, we used the sequential forward selection approach for MS feature selection. A filter method was used.

Given a feature set 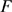 and a scoring function 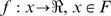, a ranking of features can be obtained computing and sorting 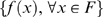. Let such ordered set be: 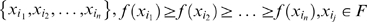.

Instead of producing all possible subsets of 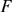, we produce the sets 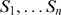 such that:
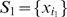


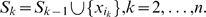



Substituting 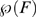 with 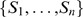 in the exhaustive approach completes the definition of the heuristic approach.

We used this method with two different rankings, respectively based on the number of mutations recorded for each codon (feature); and on the sum of the first 11 Principal Components (PCs) coefficients of each feature (where 11 was the number of PCs contributing 100% of the data variance). Panels A and B of Figure 3 report the 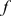 values obtained for the two different ranking functions. Panel C of the same Figure compares the best feature sets (minimal stable subsets giving 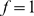).

### Geographic visualization of feature relevance

Geographic visualizations of the most relevant features of the MS, MT and FP datamatrices were obtained by respectively summing feature frequencies (for MS and MT) and *per caput* supply of each food group expressed as % of the total available calories, as detailed above [50], [17] (for FP). Resulting values were projected into yellow to red color range onto the geographic profiles of the countries and geographic areas contributing to the *TP53* mutation database.

### Correlation analyses

To perform a multivariate correlation analysis between the PCs of MS, MT and FP, we exploited their projections on the respective PCs. Both the Scree and the Kaiser [116] tests provided clear support for extracting the first 11 components for MS. When applied to MT, these tests supported the extraction of 4 and 3 PCs respectively, being the eigenvalue of the fourth PC near the lower limit value (*i.e*., 0.9). For FP the Scree and Kaiser tests indicated 3 and 4 PCs, respectively. Pairwise Pearson correlations were then computed between the PCs in all the projected spaces.

## Supporting Information

File S1 Coefficient loadings of the three most relevant PCs of mutation sites (MS), mutation types (MT) and food patterns (FP) and Pearson's correlation scores computed between the PCs of MS, MT and FP.
(0.04 MB DOC)

Figure S1 Coefficient loadings of the first three PCs of the mutation sites, mutation types and food patterns datamatrices. Coefficient loadings of the three most relevant principal components (PCs) of the mutation sites (MS, A–C), mutation types (MT, D–F) and food availability patterns (FP, G–I) datamatrices are projected on their 1-dimensional space (see File S1 for discussion).
(0.71 MB TIF)

Figure S2 Assignment of Japan to clusters I or II in cluster analysis for food availability patterns. The food category “cereals” determined clusterization of Japan with Western countries for food availability patterns. Histograms visualize the number of times that each of the 13 features was present in the 2,405 clusterings classified as type A, i.e, where Japan joined Iran and South and East Asia in cluster II-FP (A), or in the 4,178 clusterings classified as type B, i.e, where Japan joined Western countries in cluster I-FP (B). It is readily evident that feature 3 (cereals) was almost always absent in type A clusterings and almost always present in type B clusterings. This reflects the estimated low mean per caput supply of cereals available for human consumption in Japan, compared to the countries/geographic areas in the II-FP cluster (i.e., Iran and South and East Asia). Features 1 to 13 represent the following food categories: 1, animal fats; 2, animal products; 3, cereals; 4, fish/seafood; 5, fruit; 6, meat; 7, milk; 8, oilcrops; 9, pulses (legumes); 10, starchy roots; 11, sweeteners; 12, vegetable oils; 13, vegetables.
(0.15 MB TIF)

Dataset S1 1990 Food Balance Sheet data - Estimated mean per caput supply of each major food group available for human consumption in the *TP53* database countries, as extracted from the Food Balance Sheets (FBS) of the Food and Agriculture Organization of the United Nations (FAO), year 1990.
(0.02 MB XLS)

Datamatrices S1 Normalized datamatrix for *TP53* mutation sites (MS) and mutation types (MT) assigned to 12 countries/geographic areas and normalized datamatrix for the estimated food availability patterns (FP) of the 12 countries/geographic areas in the *TP53* database. Data from 2,572 *TP53* exons 5–8 mutations associated with primary CRCs were retrieved from the *TP53* database of the International Agency for Research on Cancer (IARC), R10 update, July - 2005, and from Mahdavinia et al., J Cell Physiol. 2008; 216(2):543–550.
(0.07 MB XLS)

Database S1 List of 2,572 *TP53* exons 5–8 mutations reported in association with primary colorectal cancers, including 2,475 from 11 countries/geographic areas, extracted from the *TP53* database of the International Agency for Research on Cancer (IARC), R10 update, July 2005, and 97 from Iran (Mahdavinia et al., J Cell Physiol. 2008;216:543–550).
(0.61 MB XLS)
