# Supplementary material for: Transitions at CpG Dinucleotides, Geographic Clustering of TP53 Mutations and Food Availability Patterns in Colorectal Cancer
Source: PLoS One. 2009 Aug 31;4(8):e6824. doi: 10.1371/journal.pone.0006824 (PMC2730577; doi:10.1371/journal.pone.0006824)
Supplement: File S1 — Coefficient loadings of the three most relevant PCs of mutation sites (MS), mutation types (MT) and food patterns (FP) and Pearson's correlation scores computed between the PCs of MS, MT and FP. (0.04 MB DOC) [file pone.0006824.s001.doc]

# File S1

Coefficient loadings of the three most relevant principal components (PCs) of mutation sites (MS), mutation types (MT) and food availability patterns (FP) projected on their 1-dimensional space are shown in Figure S1.

Highest coefficients in loading order (absolute values) for MS corresponded to: codons 175, 248, 273 and 245 in the first PC (A); codons 273 and 282 in the second PC (B); codons 213 and 245 in the third PC (C). For MT the highest coefficient (absolute value) in the first PC corresponded to G:C>A:T at CpG, followed in loading order (with much lower absolute values) by G:C>A:T and A:T>C:G (D). The second and third PCs for MT (that accounted for a minor fraction of variance) showed highest absolute values respectively for G:C>A:T and G:C>T:A in the second PC (E), and for frameshifts (FS) and G:C>C:G in the third PC (F). For FP the highest coefficient (absolute value) in the first PC corresponded to cereals, followed (with lower absolute values) by meat, milk, sweeteners, animal fats. The second and third PCs for FP, that together accounted for a minor fraction of variance (12.7%), showed highest absolute values respectively for vegetable oils, animal fats and milk (H) and meat (I). Pearson’s correlation between the PCs for MT and FP (see also Table 3) showed that the first PC for MT (A) was correlated with the first PC for FP (G), with a value of r=-0.60 (*P* =0.039). Due to the negative value of the correlation, cereals (G, positive value) were inversely correlated to G:C>A:T at CpGs (D, positive value) and directly correlated to G:C>A:T at non-CpGs (negative value). Meat, milk, sweeteners and animal fats (G, negative values) were directly correlated to G:C>A:T at CpGs (D, positive value). Notably there was a strong correlation between the first PCs of MS (A) and MT (D), with a value of r = -0.87 (*P* = 0.0002). Mutations at hotspot codons 175, 248, 273 and 275 (A, negative values) were directly correlated to to G:C>A:T at CpGs (D, positive value) and inversely to G:C>A:T and A:T>C:G (D, negative values). Other correlations involved second and third PCs. The first PC of MT (D) correlated with the second PC of FP (H) with a value of r= -0.60 (*P* =0.038). G:C>A:T at CpGs (D, positive value) were directly correlated to animal fats and milk (H, negative value) and inversely to vegetable oils (H, positive value). The third PC of MT (F) correlated with the first PC of FP (G), with r= 0.62 (*P*= 0.032). G:C>C:G transversions (F, negative value) were inversely correlated to cereals (G, positive value) and directly to meat, milk, sweeteners and animal fats (G, negative values). Frameshifts (FS, F, positive value) were directly related to cereals (G, positive value) and inversely to meat, milk, sweeteners and animal fats (G, negative values). The second PC of MT (A) correlated with the third PC of FP (I), with a value of r= 0.60 (*P*=0.0371). Meat (I, positive value) was directly related to G:C>T:A transversions (E, positive value) and inversely to G:C>A:T at non CpGs (E, negative value), which could be consistent with the roles of 7,8-dihydro-8-oxoguanine (8-oxo-G), a typical inflammatory base lesion, and/or of exposure to bulky electrophiles, in G>T mutagenesis [1], [2], [3], [4], [5], [6], [7], [8].

**References**

1. Marnett LJ (2000) Oxyradicals and DNA damage. Carcinogenesis 21: 361-370.
2. Kreutzer DA, Essigmann JM (1998) Oxidized, deaminated cytosines are a source of C > T transitions in vivo. Proc Natl Acad Sci USA 95: 3578-3582.
3. Kennedy LJ, Moore K Jr, Caulfield JL, Tannenbaum SR, Dedon PC (1997) Quantitation of 8-oxoguanine and strand breaks produced by four oxidizing agents. Chem Res Toxicol 10: 386-392.
4. Cheng KC, Cahill DS, Kasai H, Dosanjh MK, Singer B, et al. (1992) 8-Hydroxyguanine, an abundant form of oxidative DNA damage, causes G>T and A>C substitutions. J Biol Chem 267: 166-172.
5. Cross AJ, Sinha R (2004) Meat-related mutagens/carcinogens in the etiology of colorectal cancer. Environ Mol Mutagen 44: 44-55.
6. Chen JX, Zheng Y, West M, Tang MS (1998) Carcinogens preferentially bind at methylated CpG in the p53 mutational hot spots. Cancer Res 58: 2070-2075.
7. You YH, Halangoda A, Buettner V, Hill K, Sommer S, et al. (1998) Methylation of CpG dinucleotides in the lacI gene of the Big Blue transgenic mouse. Mutat Res 420: 55-65.
8. Denissenko MF, Chen JX, Tang MS, Pfeifer GP (1997) Cytosine methylation determines hot spots of DNA damage in the human P53 gene. Proc Natl Acad Sci USA. 94: 3893-3898.
