# Supplementary figures and images for: Transitions at CpG Dinucleotides, Geographic Clustering of TP53 Mutations and Food Availability Patterns in Colorectal Cancer
Source: PLoS One. 2009 Aug 31;4(8):e6824. doi: 10.1371/journal.pone.0006824 (PMC2730577; doi:10.1371/journal.pone.0006824)

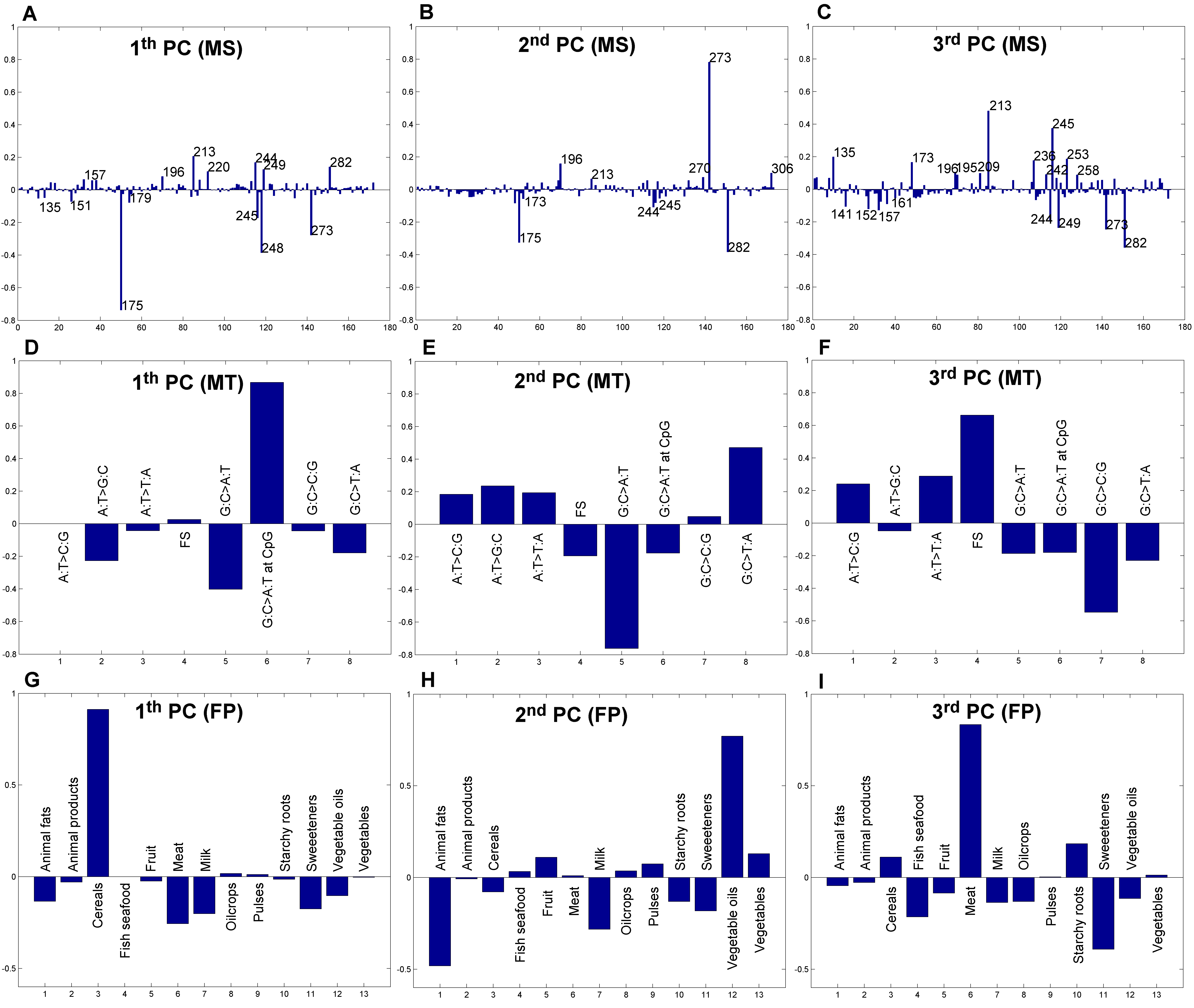

Supplement: Figure S1 — Coefficient loadings of the first three PCs of the mutation sites, mutation types and food patterns datamatrices. Coefficient loadings of the three most relevant principal components (PCs) of the mutation sites (MS, A–C), mutation types (MT, D–F) and food availability patterns (FP, G–I) datamatrices are projected on their 1-dimensional space (see File S1 for discussion). (0.71 MB TIF) [file pone.0006824.s002.tif]

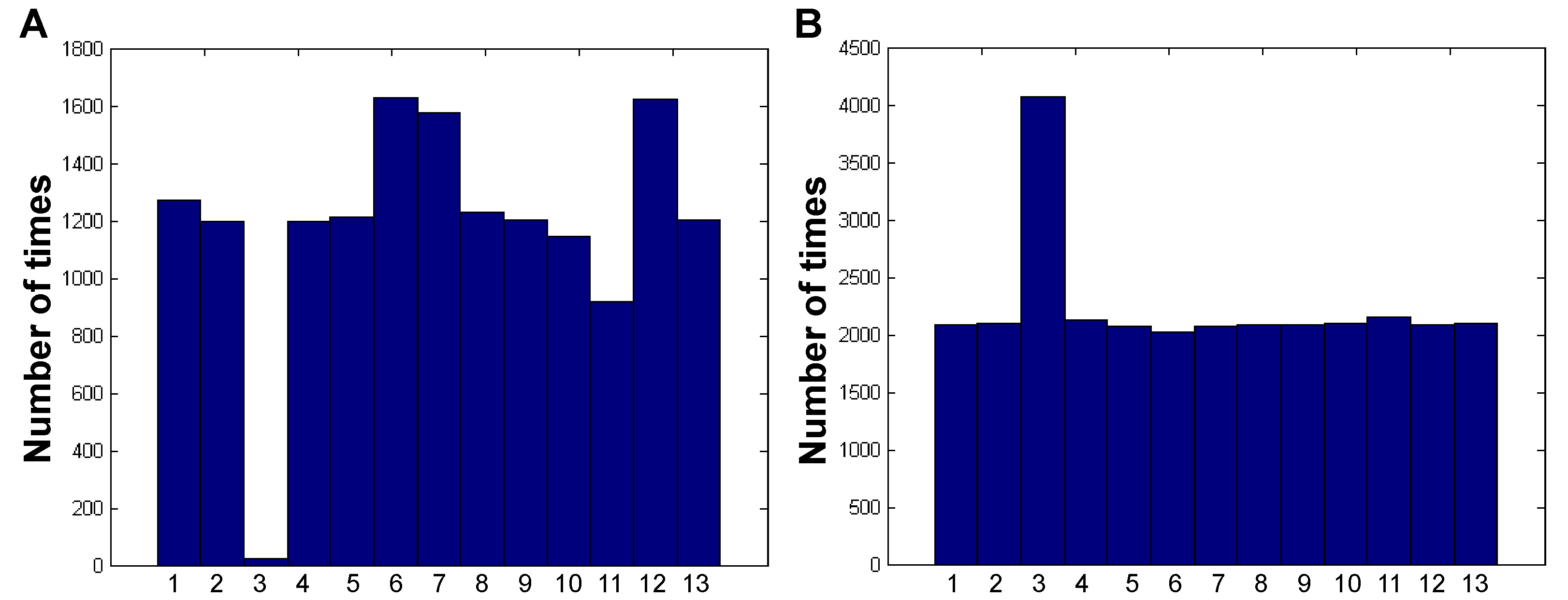

Supplement: Figure S2 — Assignment of Japan to clusters I or II in cluster analysis for food availability patterns. The food category “cereals” determined clusterization of Japan with Western countries for food availability patterns. Histograms visualize the number of times that each of the 13 features was present in the 2,405 clusterings classified as type A, i.e, where Japan joined Iran and South and East Asia in cluster II-FP (A), or in the 4,178 clusterings classified as type B, i.e, where Japan joined Western countries in cluster I-FP (B). It is readily evident that feature 3 (cereals) was almost always absent in type A clusterings and almost always present in type B clusterings. This reflects the estimated low mean per caput supply of cereals available for human consumption in Japan, compared to the countries/geographic areas in the II-FP cluster (i.e., Iran and South and East Asia). Features 1 to 13 represent the following food categories: 1, animal fats; 2, animal products; 3, cereals; 4, fish/seafood; 5, fruit; 6, meat; 7, milk; 8, oilcrops; 9, pulses (legumes); 10, starchy roots; 11, sweeteners; 12, vegetable oils; 13, vegetables. (0.15 MB TIF) [file pone.0006824.s003.tif]
